# Supplementary material for: Effect of Camel Milk on Glucose Homeostasis in Patients with Diabetes: A Systematic Review and Meta-Analysis of Randomized Controlled Trials
Source: Nutrients. 2022 Mar 15;14(6):1245. doi: 10.3390/nu14061245 (PMC8954674; doi:10.3390/nu14061245)
Supplement: Supplementary file 1 [file nutrients-14-01245-s001.zip › nutrients-1610372-supplementary.pdf]

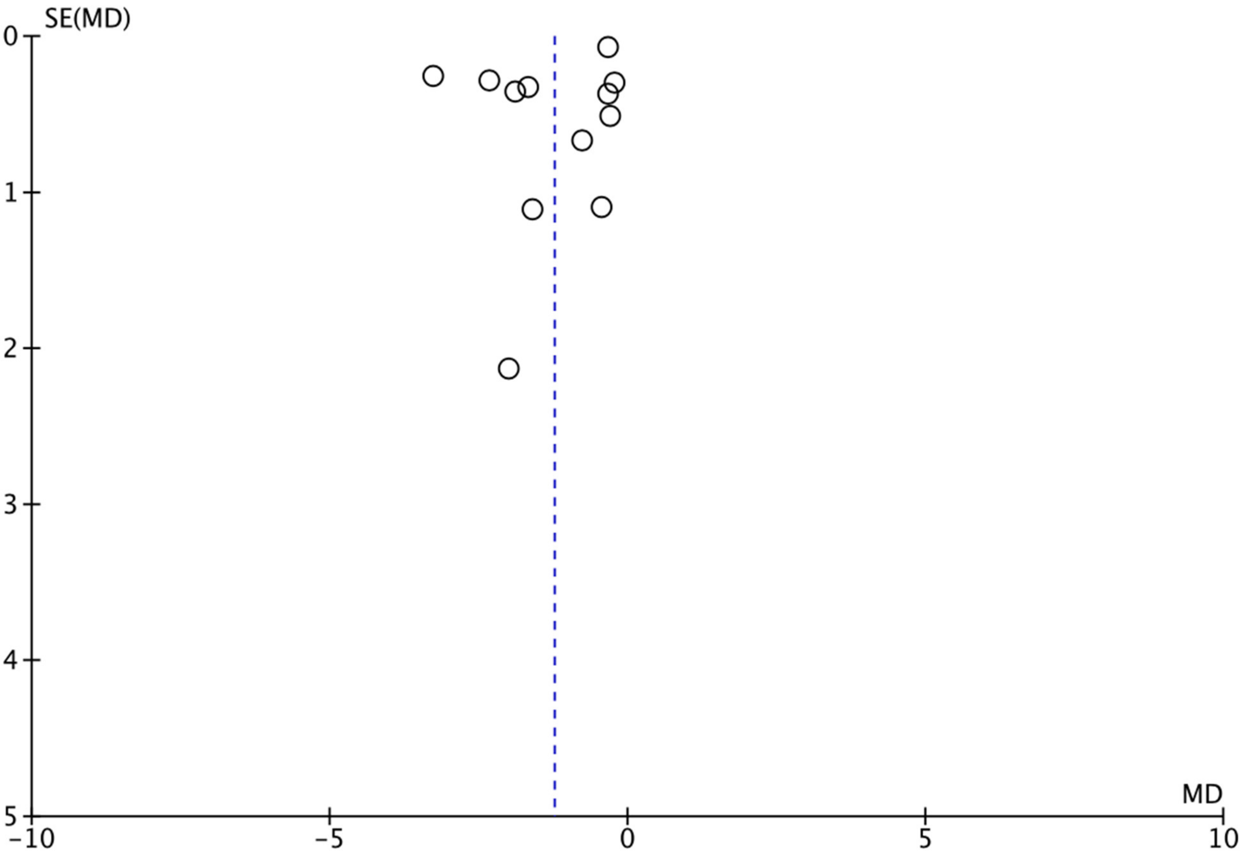

Figure S1. Funnel plot for HbA1c.

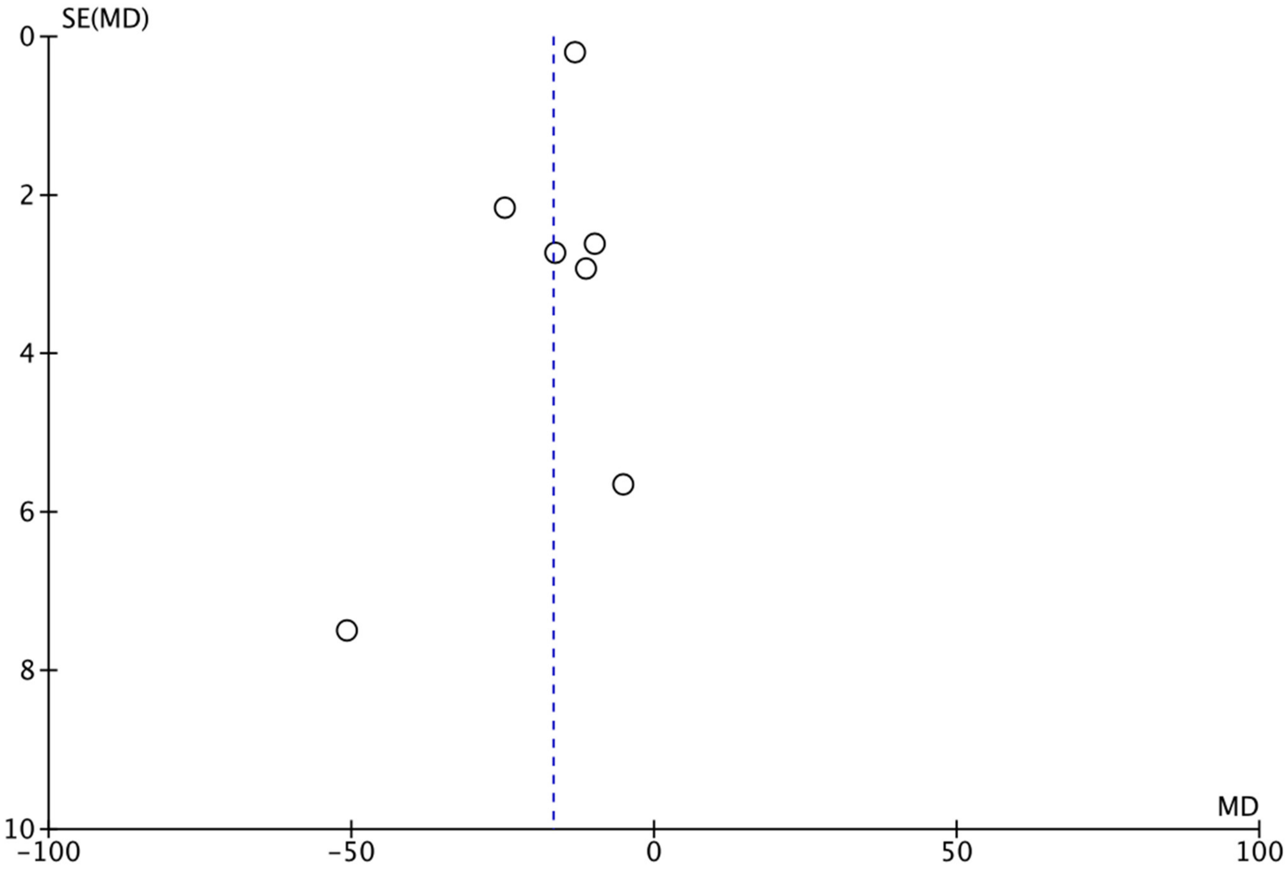

Figure S2. Funnel plot for ID.

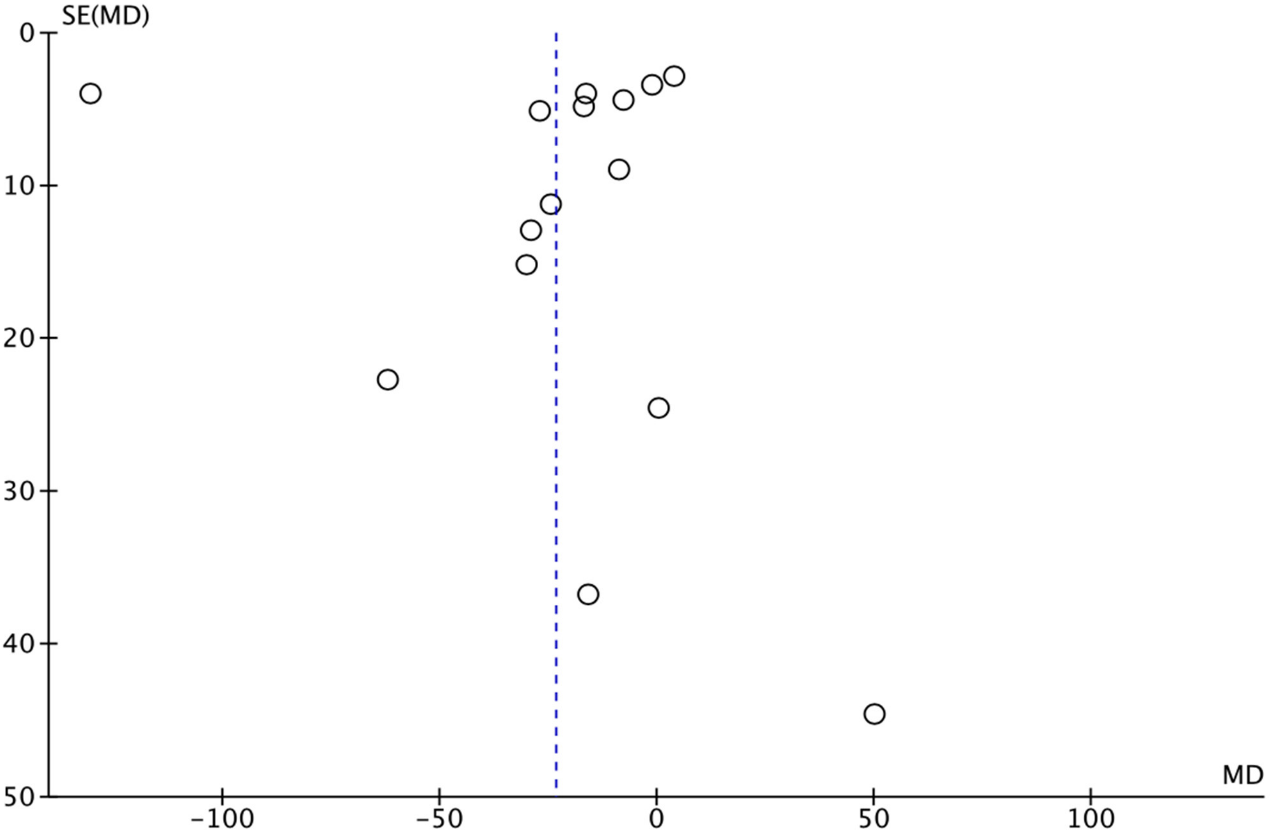

Figure S3. Funnel plot for FBG.

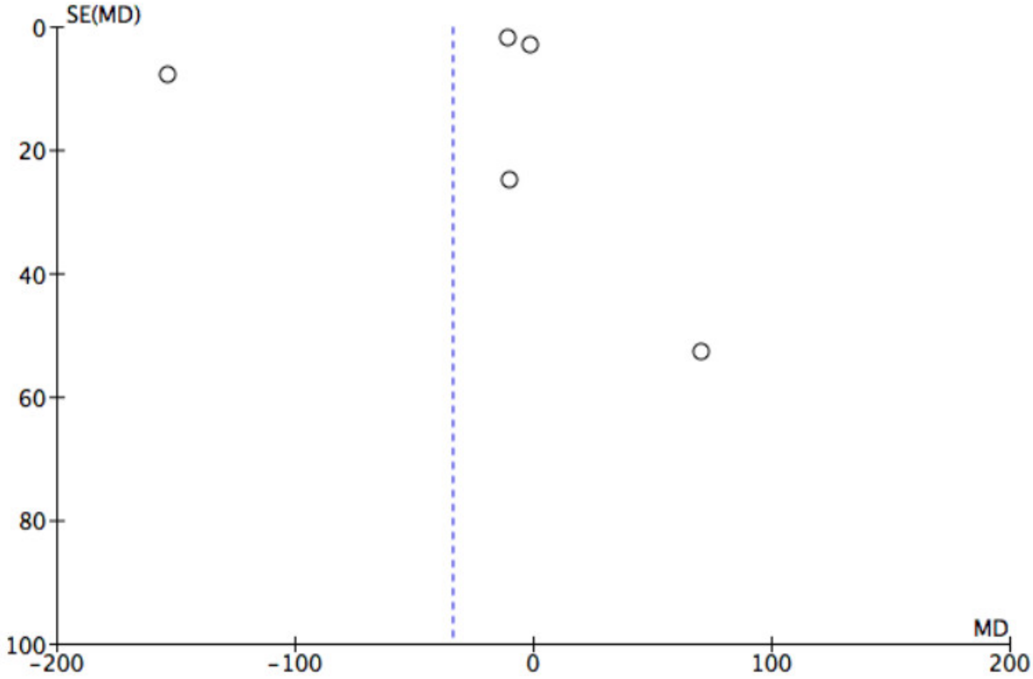

Figure S4. Funnel plot for PBG.

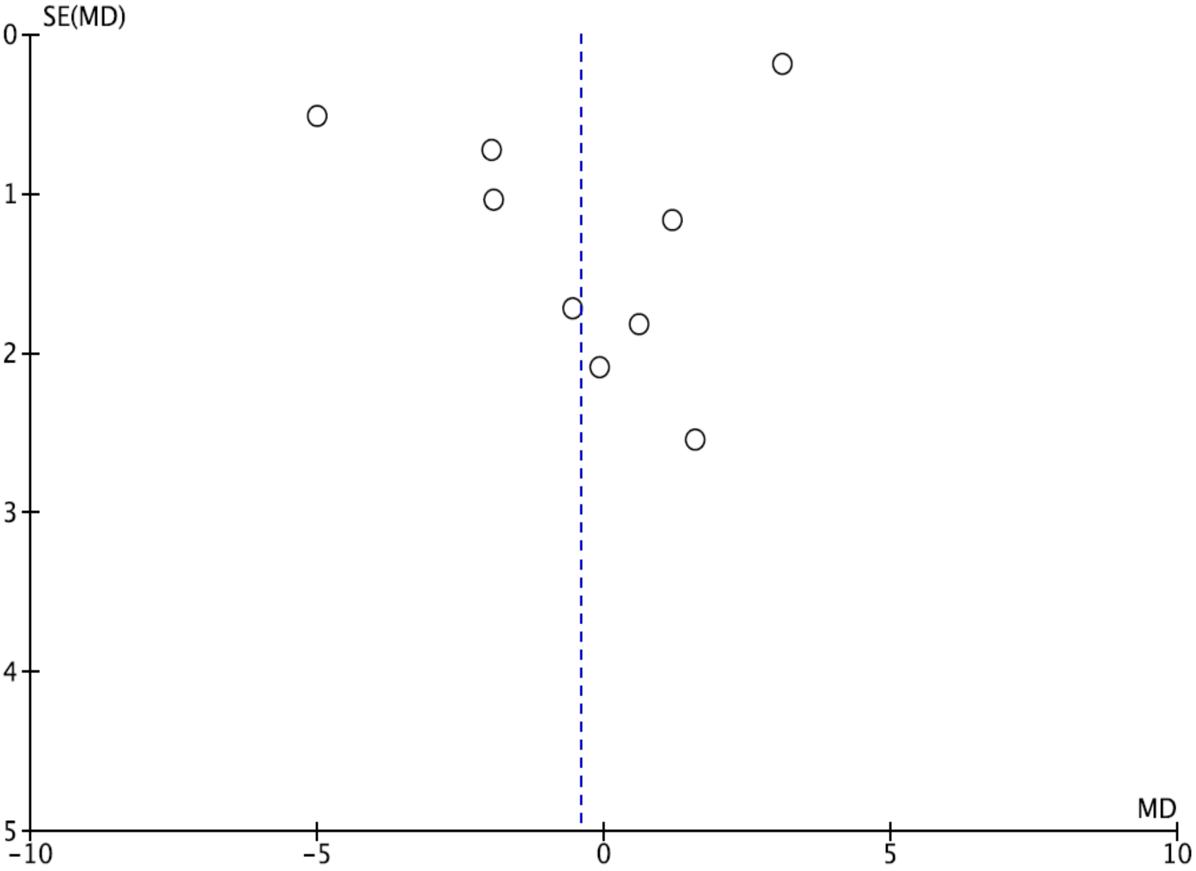

Figure S5. Funnel plot for FI.

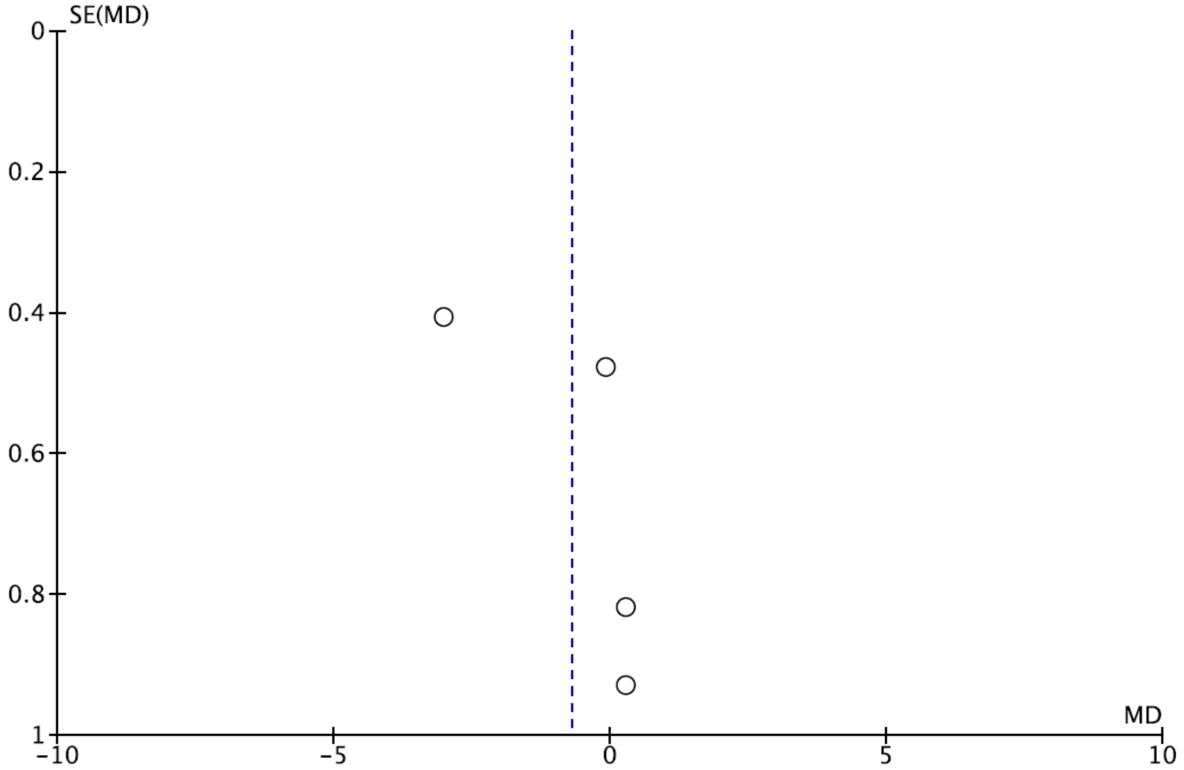

Figure S6. Funnel plot for HOMA-IR.

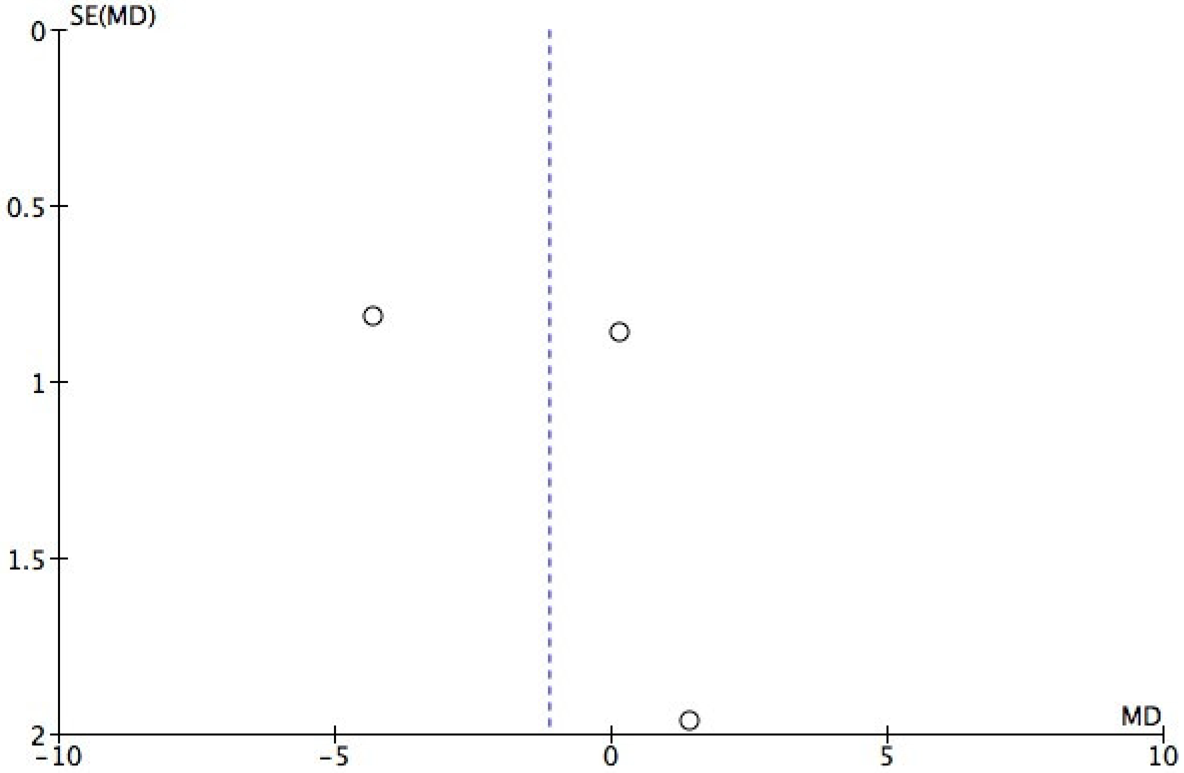

Figure S7. Funnel plot for IA.

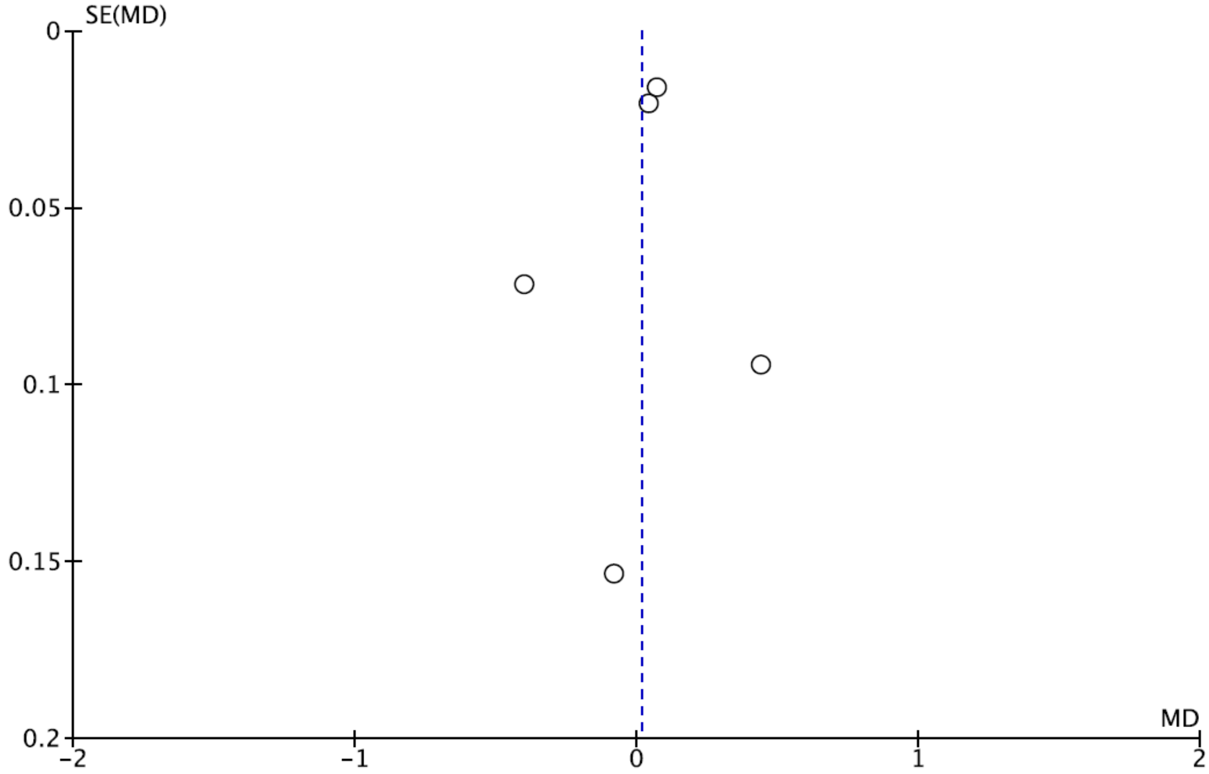

Figure S8. Funnel plot for CP.
